# Supplementary material for: Modulation in alpha band activity reflects syntax composition: an MEG study of minimal syntactic binding
Source: Cereb Cortex. 2022 Mar 21;33(3):497–511. doi: 10.1093/cercor/bhac080 (PMC9890467; doi:10.1093/cercor/bhac080)
Supplement: HardyEtAl_SupplementaryMaterials_CerebralCortex_bhac080 [file hardyetal_supplementarymaterials_cerebralcortex_bhac080.pdf]

SUPPLEMENTARY MATERIALS

Modulation in Alpha Band Activity Reflects Syntax Composition: An MEG Study of Minimal Syntactic Binding

Authors: Sophie M. Hardy, Ole Jensen, Linda Wheeldon, Ali Mazaheri, Katrien Segaert

Contact: sophie.hardy@warwick.ac.uk; k.segaert@bham.ac.uk

SUPPLEMENTARY MATERIALS 1

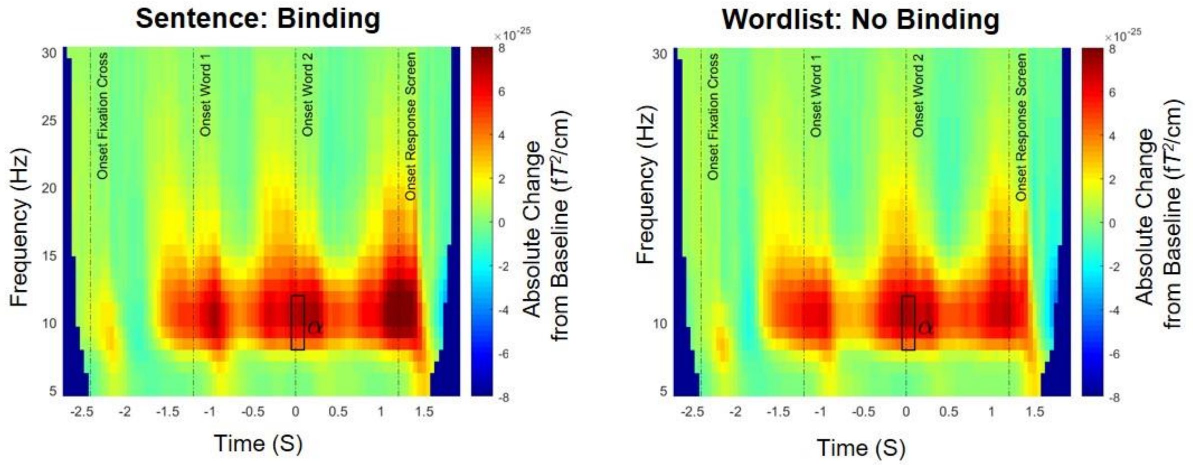

Supplementary Figure 1. Time-frequency representations (TFRs) of power averaged across all sensors for the Sentence condition [left panel], in which syntactic binding occurred (e.g., *"she grushes"*) and the Wordlist condition [right panel], in which no binding occurred (e.g., *"cugged grushes"*). Power is expressed as an absolute change from the baseline period (-2.1s to -1.6s), which occurred during the fixation cross presentation. The complete epoch is shown. Time relates to the main time period of interest, epoched around the onset of the second word (presented at 0s). We analysed both our time period of interest (-0.5s to 1s of the epoch) and the wider timeframe of the auditory stimuli presentation (-1.3s to 1.2s). The rectangle highlights the time period showing a significant difference in alpha power (8-12 Hz) between the two conditions (-0.05s to -0.1s; $p < .05$). We did not observe any other significant effects in our time-frequency analyses.

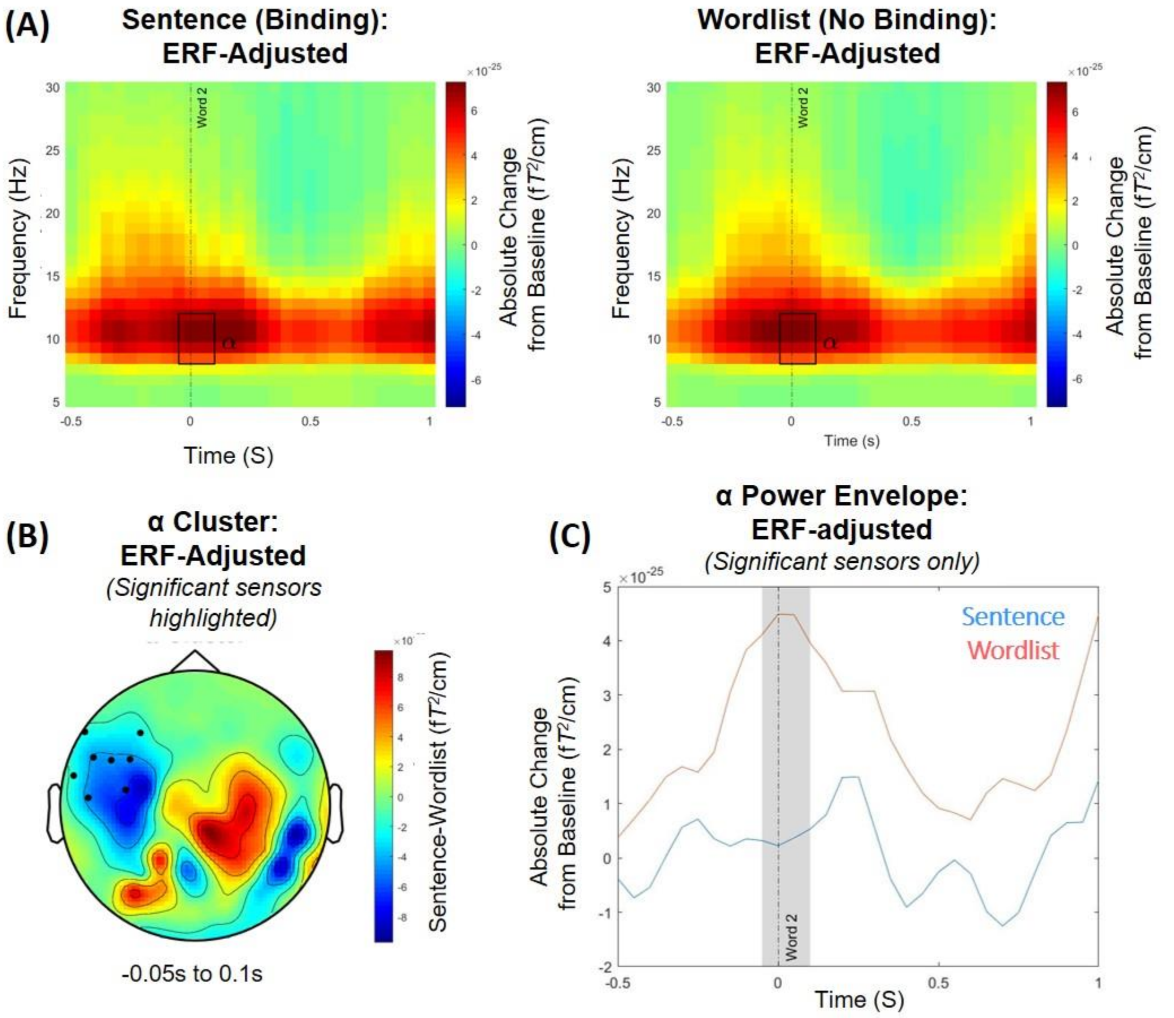

Supplementary Figure 2. To ensure that the observed oscillatory changes were not just the spectral representation of the event-related fields (ERFs), the ERF components were subtracted from the time-frequency representation of the oscillatory data (Mazaheri & Picton, 2005). The subtraction was achieved by first generating the time frequency decomposition of the ERF data for each condition and participant separately. Next, the time frequency power spectra of the ERF were subtracted from the time frequency power spectra of the MEG signal for each condition. The subsequent power changes in the time-frequency domain were used to generate time frequency power spectra differences between experimental conditions (Sentence vs. Wordlist). We then reanalysed the ERF-adjusted TRFs of power using the same statistical methods outlined in the primary time-frequency analyses. In these ERF-adjusted analyses, we found the same significant alpha power cluster at -0.05s to 0.1 (p = .022). This demonstrates that our observed oscillatory effect of alpha was distinct from the evoked fields.

Shown in this figure is: **(A)** TFRs of the ERF-adjusted power averaged across all sensors for the sentence condition [left panel] in which syntactic binding was highly to occur (e.g., *"she grushes"*), and the wordlist condition [right panel] in which binding was highly unlikely (e.g., *"cugged grushes"*); **(B)** the scalp topography of the condition contrast (Sentence-Wordlist) of the averaged alpha power activity in the time window showing a significant difference in power between conditions; and **(C)** the time course of the alpha power envelope for the sensors showing a significant difference in power between the Sentence (blue) and Wordlist (red) conditions.
